# Genotype by environment interaction and yield stability of cowpea (*Vigna unguiculata* (L.) Walp.) genotypes in moisture limited areas of Southern Ethiopia

**DOI:** 10.1016/j.heliyon.2022.e09013

**Published:** 2022-02-24

**Authors:** Yasin Goa, Hussein Mohammed, Walelign Worku, Elias Urage

**Affiliations:** a Southern Agricultural Research Institute, P. O. Box 79, Areka, Ethiopia; b Hawassa University, School of Plant and Horticultural Sciences, P. O. Box 5, Hawassa, Ethiopia; c Southern Agricultural Research Institute, P. O. Box 6, Hawassa, Ethiopia

**Keywords:** Cowpea, Biplot, Genotype by environment interaction, Yield stability

## Abstract

Genotype by environment interaction (GEI) markedly influences the success of breeding strategies in a versatile crop such as cowpea (Vigna unguiculata (L.) Walp.). Twenty cowpea genotypes were tested in a randomized complete block design with three replications at Gofa, Kucha, and Humbo in Meher seasons of 2016 and 2017 (E1 to E6) and Belg seasons of 2017 and 2018 (E7 to E12) to quantify and evaluate the effects of genotypes, environments and their interactions for grain yield of cowpea genotypes and to identify stable and/or high-yielding genotypes. The environment, genotype, and GEI effects were highly significant (p < 0.001), with the contribution of 42.3%, 23.0%, and 34.7%, respectively to the TSS. Additive main effect and multiplicative interaction (AMMI), genotype main effects plus genotype-environment interaction (GGE), ASV (AMMI stability value), and Genotype stability index (GSI) were used to identify stable genotypes. The GGE-biplot model showed that the twelve environments used for the study clustered under three mega-environments. Our results showed that IT96D-604(G12), IT-89KD (G16), IT93K-293-2-2 (G14), 93K-619-1(G13), IT97K-569-9(G20), and IT99K-1060(G15) scored the highest grain yield (1.67, 1.62, 1.55, 1.51, 1.51, and 1.45 t ha^−1^), respectively, over environments. AMMI and GGE biplots analyses identified G16 (IT-89KD) and G14 (IT93K-293-2-2) as stable and high-yielding genotypes across environments and can be further tested in variety verification and later on released as varieties and can also be used for different breeding purposes in all cowpea growing areas in southern Ethiopia. The four high-yielding genotypes IT96D-604, 93K-619-1, IT97K-569-9, and IT99K-1060 could be recommended to be included in breeding or variety verification trials for release. Moreover, our results denoted the effectiveness of AMMI and GGE biplot techniques for selecting stable genotypes, high yielding, and responsive.

## 1 Introduction

Cowpea (Vigna unguiculata (L.) Walp.) is cultivated on about 12 million hectares globally where over 95% (or 11.4 million ha) of which are grown in Sub-Saharan Africa (FAOSTAT, 2015) with amounted production 4.46 million metric tons of which total production share for Africa was estimated at 4.24 million metric tons (FAO, 2015). Concerning its consumption, 52% for food, 13% as animal feed, 10% for seeds, 9% for other uses, and 16% are wasted (Baysah, 2013). Cowpea is a vital food legume in many African countries, including Ethiopia where tender leaves, young/fresh pods, and grains are used for human food, while the foliages are an important livestock feed (Olawale and Bukola, 2016; Mulugeta et al., 2016). It is an inexpensive source of protein and is used as an excellent substitute for animal proteins by many low-income Africans in the low-land humid and dry savannah tropics (Nwosu et al., 2013). Cowpea is primarily used for human food in the form of boiled grains (Nifro), bread (Kita) and as a constituent for various sauces like Shiro *wot* in northern Ethiopia (Mulugeta et al., 2016).

Cowpea does well and is most popular in the semiarid parts of the tropics where other food legumes do not perform well (Sankie et al., 2012). It is a hardy crop and well adapted to drought-prone areas (Badiane et al., 2012). It has deep roots and a dense foliage cover that not only stabilizes the soil but also protects the ground and retains moisture. These traits are of particular importance in the dry zones where moisture is limited (NRC, 2006). The environmental advantage of cowpea is also arising from its ability to cope in semi-arid regions with low external inputs (Boukar et al., 2018). Like other legumes, cowpea also fixes atmospheric nitrogen, compensating for the loss of nitrogen absorbed by cereals, replenishing the nitrogen status of the soil, and restores soil fertility (Tesema and Esthetayehu, 2003; Sanginga et al., 2003).

In Ethiopia, cowpea is grown in the drier areas of Oromiya (the Rift Valley, highlands of Hararge), Amhara (Shewarobit, Kobo and Waghimira areas), South Nation, Nationalities, and Peoples Regional State (SNNPRS) (Konso, Derashe, Humbo, Hammer Bako, Loka Abaya, Gofa, and Loma woredas and South Omo zone), Tigray, and Gambella (Kassaye et al., 2013; Tesema and Esthetayehu, 2003). Nearly 70% of the arable land in Ethiopia falls on dry land environments where rainfall is usually inadequate, poorly distributed, and varies over years and seasons within a year (UNDP, 2014). Despite its significance and production, cowpea has been identified as a neglected and underutilized crop species, with further research required in some parts of Africa including Ethiopia where there is limited information on its cultivation, agronomic practices, and seed handling (Chivenge et al., 2015; Mfeka et al., 2019; Selamawit et al., 2020). In Ethiopia, only six varieties have been released so far (Ministry of Agriculture, 2018). Statistical data on cowpea area and production are not available in the country which usually is reported with that of haricot beans (Phaseolus vulgaris L.). The average national cowpea yield in the farmers’ field in Ethiopia is estimated to be 0.4 t ha^−1^ (Beshir et al., 2019), a range far below the average yield of 2.2–3.2 t ha^−1^ recorded in research farms with proper crop management and protection practices (Ashinie et al., 2020, Ashinie et al., 2020). The low average national yield is often associated with the use of unimproved local varieties, poor soil fertility, biotic and abiotic stresses, and inadequate technological interventions.

There is an urgent need to increase cowpea yields by developing superior genotypes in the South region, as it is one of the major production complexes in the country (Beshir et al., 2019). Several authors have reported inconsistency in the performance of cowpea genotypes over different environments or failure of genotypes to achieve the same relative performance in different environments (Baker and Léon, 1988; Ceccarelli, 1996) due to a phenomenon known as GxE Interaction (Kassaye et al., 2013; Agbahoungba et al., 2016; Odeseye et al., 2018). Genotype by environment interaction (GEI) refers to the differential performance of genotypes in different environments that affects the efficiency of selection in breeding programs (Natalia de Leon et al., 2016). Selection for yield under moisture stress is generally less effective than selection for yield under well-watered conditions due to large GEI, which notoriously hinders the identification and release of a superior crop variety (Rosielle and Hamblin, 1981). A crop variety is best when it performs consistently with a high mean yield when grown across diverse environments (Eberhart and Russell, 1966). Genotype by environment interaction (GEI) refers to the differential performance of genotypes in different environments that affects the efficiency of selection in breeding programs (Natalia de Leon et al., 2016). Knowledge of the magnitude and pattern of GEI and stability analysis is important for understanding the response of different genotypes to changing environments and for identifying stable and widely adopted and/or unstable but specifically adapted genotypes. GEI can be reduced by identifying the most stable genotypes (Eberhart and Russell, 1966; Horn et al., 2018) or by dividing the production area of the crop into mega-environments where homogenous locations can be identified. Evaluation of genotypic performances in multi-location experiments provides valuable information about the adaptation and stability of the varieties to be released (Dagnachew et al., 2014). Though many studies on other legumes have been reported for stability in southern Ethiopia (Asfaw et al., 2012); those on cowpea are relatively few. The performance of these released varieties and promising lines in the pipeline has not been studied under the moisture limited areas. There is also limited information on the extent and pattern of GxE interaction and the stability of these genotypes when grown in the region. Therefore, the objectives of the present study were to assess the magnitude and pattern of GxE interactions and to assess the stability of the cowpea genotypes to identify stable and high-yielding varieties for broad or narrow adaptation to enhance productivity in southern Ethiopia.

## 2 Materials and methods

### 2.1 Experimental sites

**Figure 1:**
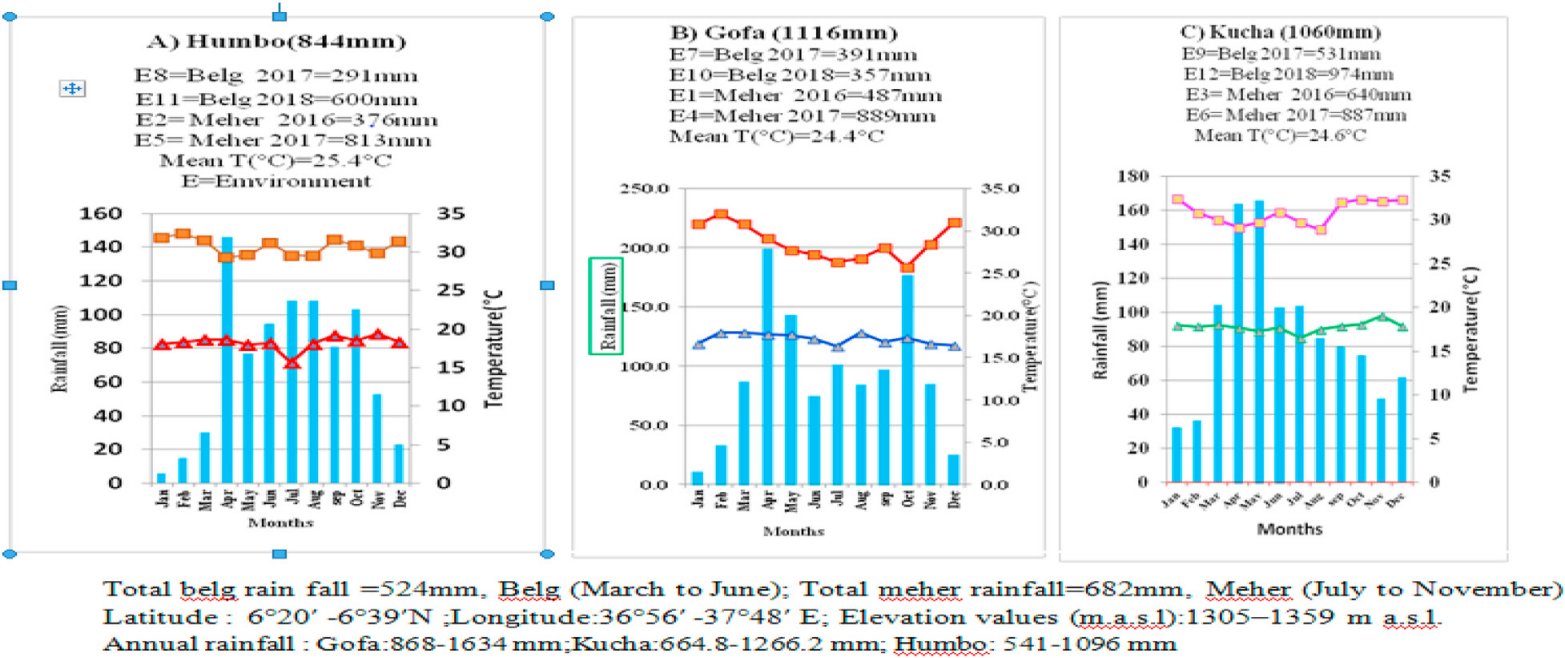
Monthly rainfall, minimum and maximum temperature at the three-test sites. (Source: Ethiopian Meteorology Agency, Awassa branch, 2018).

Field experiments were carried out in the ‘Meher’ in 2016 and 2017(E1 to E6) and the ‘Belg’ in 2017–2018 (E7 to E12) cropping seasons at three (Gofa, Kucha, and Humbo) in southern Ethiopia. Experiments conducted during Meher of 2016 were designated as E1-E3; those conducted during Meher of 2017 were designated as E4-E6. The experiments of Belg 2017 were designated as E7-E9 while those of Belg 2018 were named E10-E12 (Figure 1). The study sites are located in three districts (Gofa, Kucha and Humbo) of the south region (6°20′ -6°39′N; 36°56′ -37°48′ E) at a low altitude range of 1305–1359 m a.s.l. (Figure 1). These three locations are well-representing moisture-limited agro-ecologies with substantial cowpea production in southern Ethiopia. The soil type comprises sandy loam in Gofa and clay in both Kucha and Humbo districts. They have a bimodal rainfall pattern with the experiments were done in Belg (short growing season from March to early June) and Meher season (main crop season which extends from July to November). In each location and season, the experiments were executed over two years with a total of 12 environments. Description of the experimental sites and meteorological data are shown in Figure 1.

At Gofa and Kucha, Belg rain started in March, while in Humbo it started in April (Figure 1). Precipitation in March at Humbo was very low. Therefore, the Belg season at Humbo was shorter than that in Gofa and Kucha. All three locations experienced maximum rainfall in April. Rains were usually irregular and come as few heavy showers. Long dry spells occurred during the growing season. Bimodality is more vivid in Gofa where rainfall in June was low (about 70 mm). However, there was a dry spell in June in all three locations. At Humbo and Kucha higher rainfall was obtained in June (90 mm and 100 mm, respectively) as compared to Gofa (70 mm). The Meher rains (July to September/October) were more even, decreasing gradually in September and October at Humbo and Kucha. At Gofa, the second-highest rainfall after April was reached in October (180 mm), with some rains (80 mm) in November, too. Gofa is lying adjacent to the lowlands of Borana in Oromiya Regional State, which are characterized by “Hagayya” rains (August to November).

### 2.2 Treatments and experimental design

Treatments consisted of twenty cowpea genotypes (five released varieties, five introduced materials, nine advanced lines, and local check) were laid out in a randomized complete block design (RCBD) with three replications. The plot size was 2.4 m wide and 3 m long with a total gross plot area of 7.2 m2. Seeds were hand planted by placing two seeds per hill at a row spacing of 0.6 m and plant spacing of 0.2 m. After emergence, the seedlings were thinned to maintain the proposed plant density per plot. Description of cowpea genotypes used in the trial is presented in Table 1. The recommended amount P in the form of di-ammonium phosphate (DAP) at 100 kg/ha was applied at planting. All other field management practices such as cultivation, weeding, etc., were carried out during the crop growing season. Aphid was controlled by spraying insecticide malathion at the rate of 1.0 L a.i/200 during seedling and pod formation stages.

**Table 1:** Cowpea Genotypes tested during 2016–2018 at 12 Environments.

No	Genotype	Genotype code	Status	Year of release	Source
1	Brazil-1	G1	Introduced	-	MARC
2	Bole	G2	Released	2005	MARC
3	White wonderer trailing	G3	Released	1976	MARC
4	Kenketi	G4	Released (standard check)	2012	MARC
5	Brazil-2	G5	Introduced	-	MARC
6	Brazil-3	G6	Introduced	-	MARC
7	Brazil-4	G7	Introduced	-	MARC
8	KB	G8	Introduced	-	MARC
9	BEB	G9	Released	1976	MARC
10	TVU-1977-DD1	G10	Released	1978	MARC
11	Local check	G11	Farmers seed (local check)	-	Farmers
12	IT96D-604	G12	Advanced line	-	MARC
13	93K-619-1	G13	Advanced line	-	MARC
14	IT93K-293-2-2	G14	Advanced line	-	MARC
15	IT99K-1060	G15	Advanced line	-	MARC
16	IT-89KD	G16	Advanced line	-	MARC
17	IT97K-499-38	G17	Advanced line	-	MARC
18	IT93K-452-1	G18	Advanced line	-	MARC
19	IT98K-1111-1	G19	Advanced line	-	MARC
20	IT97K-569-9	G20	Advanced line	-	MARC

Note: MARC = Melkasa Agricultural Research center.

### 2.3 Data collection

The observations were recorded in each genotype, in each replication, on five randomly selected plants, excluding border plants for plant height, the number of pods per plant, seeds per pod, and their mean values were used for statistical analysis. In addition, data on phenology (days to 50% flowering and maturity) and grain yield, and hundred seed weight were recorded. Grain yield samples were taken from a net plot area of 3.6 square meters adjusted to storage moisture content (10%) based on the value of actual grain moisture read by Digital Grain Moisture Meter (DRAMINSKI, POLAND). Seed weight (g) was determined by counting 100 seeds randomly from each plot yield using an electronic sensitive balance (Mark: Cosmo digital scale).

### 2.4 Data analysis

Out of the collected parameters, this paper mainly focused on grain yield data. Each location-season-year combination was considered as a separate environment in this study, producing twelve environments that were considered random. The General Linear Model (GLM) of SAS software (SAS, 2008) was used for ANOVA of data from individual locations and the combined data. Bartlett's test (1937) was used to assess the homogeneity of error variances before combined analysis was done over the twelve environments.

Stability analysis was conducted using the SAS program developed by Hussein et al. (2000). Yield stability statistics were calculated across twelve environments using AMMI Stability Value (ASV) and genotype selection index (GSI). In addition, AMMI's stability value (ASV) was calculated to rank genotypes in terms of stability using the formula suggested by Purchase et al. (2000) as shown as follows:

$$\text{AMMI}\;\text{stability}\;\text{value}\left(\text{ASV}\right)=\sqrt{{\left[\frac{\text{SSIPCA}1\left(\text{IPCA}1\text{score}\right)}{\text{SSIPCA}2}\right]}^{2}+{\left[\text{IPCA}2\;\text{score}\right]}^{2}}$$

where SSIPCA1 and SSIPCA2 are the sums of squares by the IPCA1, IPCA2, respectively, and the weight given to the IPCA1value is computed by dividing the IPCA1 sum of squares by the IPCA2 sum of squares. The larger the IPCA1 score, either negative or positive, the more specifically adapted a genotype is to certain environments. Smaller IPCA1 scores indicate a more stable genotype across environments. Similarly, the IPCA2 score near-zero revealed more stability, while large values indicated more responsive and less stable genotypes. Smaller ASV scores indicate a more stable genotype across environments. In addition, a genotype selection index (GSI) was used to determine the genotype's adaptability by combining both yield and stability in the environment/location. The GSI as described by Farshadfar (2008) was computed by the following formula: GSI = RASV + RY.

RASV is the rank of the genotypes based on the AMMI stability value; RY is the rank of mean cowpea yields in all environments (RY). GSI incorporates both mean yield and stability in a single criterion. Low values of GSI showed desirable genotypes with high mean yield and stability. GSI is relevant when looking for the most stable genotypes that do not always have the best yield performance.

Two multivariate analytical tools, AMMI (Zobel et al., 1988) and (GGE) biplots, were also used to shed more light on the significant GxE interaction and determine the stability and adaptability of each genotype.

## 3 Results and discussion

### 3.1 ANOVA of grain yield

Results of the combined analysis of variance (Table 2) revealed a significant difference among the 12 environments and also the 20 cowpea genotypes for grain yield that exhibited the presence of variability in genotypes and diversity of growing conditions at different environments. There was a significant difference among the 20 cowpea genotypes at each of the environments, pointing to the presence of wide genetic variance which can be exploited in the improvement of grain yield in the major cowpea producing areas of southern Ethiopia. The Genotype by Environment Interaction (GEI) was also significant (Table 2) which reflected the differential response of genotypes in various environments. The GEI sum of squares was about 1.5 times as large as that of the genotypes. This confirmed that GEI was highly significant and had a remarkable effect on genotypic performance in different environments. As GEI was significant, it was possible to proceed and calculate stability (Dagnachew et al., 2014). Environment explained 42.3 %, while Genotypes (G) and GEI captured 23.0 % and 34.7% of the treatment sum of square (G + E + GEI), respectively (Table 2). Therefore, the environment was the main source of variation which had a big effect on the yield of cowpea genotypes. The three locations differed in soil properties and in annual and seasonal (Belg and Meher) rainfall. The three years (2016, 2017, and 2018) during which the experiments were conducted also differed in terms of rainfall. The two seasons within each year also differed in terms of rainfall amount and distribution. Rainfalls in Belg were lower and more erratic than in Meher. All these were components of the Environment. Kuruma et al. (2019), Mohammed et al. (2017), and Yan and Kang (2003) also reported that the environment had the largest effect on grain yield.

**Table 2:** Combined and AMMI ANOVA of Grain Yield of 20 Cowpea Genotype grown across 12 Environments.

Source of variation	DF	SS	MS	VE (%)	% GEI	Cumulative (%)
Treatments (G + E + GEI)	239	153087843	640535∗∗∗			
Genotypes (G)	19	35204932	1852891∗∗∗	23.0		
Environments (E)	11	64756683	5886971∗∗∗	42.3		
Block	24	785002	32708			
Interactions(GEI)	209	53126227	254192∗∗∗	34.7		
IPCA 1	29	17602987	607000∗∗∗		33.1	33.1
IPCA 2	27	13277103	491745∗∗∗		25.0	58.1
IPCA 3	25	5747041	229882∗∗∗		10.8	68.9
IPCA 4	23	4625852	201124∗∗∗		8.7	77.6
Residuals	105	11873245	113079		22.3	100.0
Error	456	15348006	33658			

Note: DF = Degrees of freedom, SS = Sum of squares, MS = Mean Squares, VE (%) = variation explained as % of Treatment SS, % GEI = Percentage of genotype by environment interaction sum of Squares, IPCA = Interaction Principal Component Axis. ∗∗∗, significant at 0.01 probability level.

### 3.2 Comparison of mean grain yields

The big environmental effect for grain yield indicated that environments were diverse with the large differences among environmental means. The mean grain yield of the environments ranged from 0.82 t ha^−1^ (E11, Humbo, Belg, 2018) to 1.88 t ha^−1^ (E4, Gofa, Meher, 2017) (Table 3). E2, E8, and E11 (all at Humbo) yielded below the grand mean of 1.3 t ha^−1^. Only one environment of Humbo (E5, Meher, 2017) yielded higher than the grand mean. E4 (Gofa, Meher, 2017) and E6 (Kucha, Meher, 2017) yielded 1.88 t ha^−1^ and 1.641 t ha^−1^, respectively. Except for E12 (Kucha, Belg, 2018, 1.03 t ha^−1^), all environments of Gofa and Kucha yielded above-average grain yield.

**Table 3:** Mean grain yield (t ha^−1^) of 20 cowpea genotypes at 12 environments.

Code	E1	E2	E3	E4	E5	E6	E7	E8	E9	E10	E11	E12	GM
G1	1.12	0.92	0.87	1.53	0.87	1.27	0.80	0.44	1.31	1.21	0.45	0.39	0.93
G2	1.53	1.22	1.31	2.27	1.40	1.79	1.52	0.54	0.88	1.77	1.02	1.10	1.36
G3	1.70	1.62	1.78	1.42	1.21	1.26	1.64	0.92	1.48	1.30	0.81	1.49	1.38
G4	1.37	1.55	1.03	2.08	1.53	1.61	1.58	0.65	1.48	1.72	0.94	1.12	1.39
G5	1.95	0.95	1.19	2.08	1.59	1.64	0.92	0.91	1.48	1.71	0.69	0.63	1.31
G6	1.98	0.72	0.99	2.21	1.97	1.84	1.22	0.94	1.32	1.66	0.59	0.45	1.32
G7	1.92	1.40	1.21	1.57	1.30	1.65	0.85	0.90	1.94	1.95	0.88	0.35	1.33
G8	1.32	0.53	0.84	1.66	0.90	1.53	0.99	1.11	0.93	1.12	0.58	0.68	1.02
G9	1.11	1.08	0.83	1.32	0.58	0.87	1.48	0.52	0.89	1.01	0.45	0.90	0.92
G10	1.68	1.31	2.00	1.46	1.06	1.61	1.52	0.93	1.24	1.48	0.86	1.36	1.38
G11	0.91	0.66	1.32	2.03	1.07	1.73	1.15	1.05	0.67	0.86	0.33	0.76	1.05
G12	1.38	1.02	2.04	2.34	1.99	2.35	1.97	1.11	2.03	1.94	1.14	0.71	1.67
G13	1.37	0.66	1.56	2.50	1.97	1.93	1.85	0.94	1.50	1.55	1.13	1.12	1.51
G14	1.44	0.87	1.16	2.46	1.57	1.74	1.84	1.25	1.97	1.78	0.90	1.67	1.55
G15	0.94	0.65	1.40	1.84	1.75	1.76	1.93	1.17	1.61	1.35	1.43	1.62	1.45
G16	1.54	1.21	1.83	2.31	1.81	1.77	1.39	1.46	1.34	1.85	1.43	1.48	1.62
G17	1.20	0.68	0.96	1.71	0.95	1.50	1.71	0.74	1.17	1.17	0.48	0.92	1.10
G18	1.19	0.80	1.05	1.37	1.12	1.41	1.73	0.68	0.91	1.02	0.50	0.97	1.06
G19	1.16	0.97	1.75	1.61	1.02	1.66	1.64	0.56	0.89	0.99	0.53	1.31	1.17
G20	1.14	0.86	1.48	1.84	1.65	1.90	1.84	1.36	1.56	1.67	1.32	1.54	1.51
EMS	0.025	0.029	0.036	0.045	0.031	0.058	0.028	0.035	0.028	0.030	0.039	0.021	-
EM	1.40	0.98	1.33	1.88	1.36	1.64	1.48	0.91	1.33	1.46	0.82	1.03	1.30
CV	11.2	11.3	11.9	11.3	17.3	12.9	14.7	14.1	12.5	23.9	22	14.2	14.2
LSD	0.26∗	0.35∗∗	0.27∗	0.28∗	0.28∗	0.29∗	0.4∗∗	0.24∗	0.28∗	0.33∗	0.33∗	0.31∗	-
	GF	HB	KU	Mean	%		GF	%	HB	%	KU	%	
Meher	1.64	1.17	1.49	1.43	**22.4**		1.64	**11.7**	1.17	**35.7**	1.49	**25.9**	
Belg	1.47	0.87	1.18	1.17			1.47		0.87		1.18		
**Mean**	**1.55**	**1.02**	**1.33**				**1.55**		**1.02**		**1.33**		

Note: GF = Gofa, HB = Humbo, KU = Kucha, EMS = Error mean square, EM = Environmental mean, Environments: E1, E2, E3 (Gofa, Humbo, and Kucha in Meher, 2016), E4, E5, E6 (Gofa, Humbo, and Kucha in Meher, 2017), E7, E8 and E9 (Gofa, Humbo, and Kucha in Belg, 2017), E10, E11 and E12 (Gofa, Humbo, and Kucha in Belg, 2018), GM = Grand Mean.

Mean grain yields were higher at Gofa (1.55 t ha^−1^) and Kucha (1.33 t ha^−1^), but low at Humbo (1.02 t ha^−1^) (Table 3). Humbo has generally lower annual rainfall (844 mm) and shorter growing season both during the Belg and the Meher seasons (Figure 1) and produced low grain yield. Gofa has a high annual mean rainfall of 1116 mm and sandy loam soils and was the most favorable location for cowpea production.

Yields were higher during the Meher season as compared to those during the Belg season by 11.7 %, 35.8 %, and 25.9%, at Gofa, Humbo, and Kucha, respectively. All Meher environments (E1 to E6), except E2, produced above-average grain yields (>1.30 t ha^−1^), while only half of the Belg environments (E7, E9, and E10) produced above-average grain yield. Rainfall was higher and the growing season was longer during the Meher season (Figure 1).

Yield responses of each genotype at different environments varied. The mean grain yield over twelve environments ranged from 0.92 (G9) to 1.67 t ha^−1^ (G12) with a grand mean of 1.3 t ha^−1^ (Table 2). Thirteen genotypes (G2, G3, G4, G5, G6, G7, G10, G12, G13, G14, G15, G16, and G20) yielded above the grand mean (1.3 t ha^−1^) and the remaining 7 were below the average yield. In addition, G12 (IT96D-604), G16 (IT-89KD), G14 (IT93K-293-2-2), G20 (IT97K-569-9), G13 (93K-619-1), and G15 (IT99K-1060) (1^st^ to 6^th^) produced the highest average yields of 1.45–1.67 t ha^−1^ over the 12 environments. These advanced lines had 4.3–20.2%, and 38.1–59.7% yield advantage over the best standard check, G4 (Kenketi), and the local check (G11), respectively. These genotypes (G12, G16, G14, G20, and G13) are recommended to be included in the breeding program or variety verification trials for release as new varieties to increase the variety portfolio of this neglected crop for which only six varieties have been released so far in Ethiopia. Genotypes G17, G18, G19, G11 (local check), G8, G1, and the released variety G9 (BEB) were the lowest yielding genotypes, ranking 16^th^ to 20^th^. Released varieties G2 (Bole), G3 (White wonderer trailing), G4 (Kenketi), and G10 (TVU-1977-DD1) ranked 10^th^, 8^th^, 7^th^, and 9^th^ by mean grain yield over environments. Similar findings on the performances of cowpea genotypes (Horn et al., 2018; Agbahoungba et al., 2016) and soybean genotypes (Asfaw et al., 2012) across environments were reported.

The strong GxE interaction showed the inconsistency of the performance of the genotypes over environments. There were large rank changes for some of the genotypes over the 12 environments. For example, genotype G6 was the highest yielding genotype at E1. However, it was one of the lowest yielding genotypes and ranked 15^th^, 16^th^, 15^th^, and 18^th^ at E2, E3, E7, and E12, respectively. G7 ranked 1^st^ at E10 but ranked 19^th^ and 20^th^ at E7 and E12, respectively. Large rank changes were also observed for G2, G3, G15, and G20. 90% of the GxE interaction was due to lack of correlations (rank change) and only 10% was due to heterogeneity of variances (analysis not shown). Matus-Cadiz et al. (2003) in wheat also observed cross-over type of GxE interaction.

These complex GxE interactions indicate the need for stability analysis which is given in the following sections. In agreement with this finding, using the AMMI model, different authors reported responses of cowpea genotypes for grain yield at varied environments (Horn et al., 2018; Kuruma et al., 2019). Several authors have also reported significant GEI and pointed to the need for stability analysis in cowpea (Kassaye et al., 2013; Agbahoungba et al., 2016).

### 3.3 Additive main effects and multiple interaction (AMMI) model

#### 3.3.1 AMMI analysis of variance

Four of the 11 IPCAs were statistically significant (Table 2). Hernandez and Crossa (2000), stated that the Gollob F-test is very liberal and can result in many multiplicative terms judged significantly. The presence of GEI was verified by the AMMI model when the interaction was partitioned among the first four principal components Axis (IPCA) as they were significant in a post-assessment. In our study, the first and second IPCAs explained 33.1 and 25.0% and cumulatively captured 58.1% of the sum of squares and 26.8% of the DF of GEI (Table 2). Though the plotting of more than two IPCA components in pairs is tedious and difficult to interpret, AMMI2 may be inadequate to explain the complex interaction of GxE in our study. The IPCA1 explained 33.1% of the interaction sum of the square in 13.87% interaction degree of freedom. Similarly, the second, third and fourth principal component Axis (IPCA 2–4) explained a further 25.0, 10.8, and 8.7 % of the GEI sum of the square, respectively. AMMI4, which cumulatively captured 77.6% total GEI SS, using 104 degrees of freedom, would be, thus, considered adequate (Table 2). This implies that the interaction of 20 genotypes of cowpea with twelve environments was predicted by the first four principal components of genotypes and environments, which is in agreement with the recommendation of Sivapalan et al. (2000). This is also following the results of Kayode et al. (2009), whereas much as the first four IPCAs were significant. However, this contradicted the findings of Zobel et al. (1988) and Abiriga et al. (2020), which recommended that the most accurate model for AMMI, can be predicted using the first two IPCAs.

#### 3.3.2 AMMI biplots (AMMI-4)

**Figure 2:**
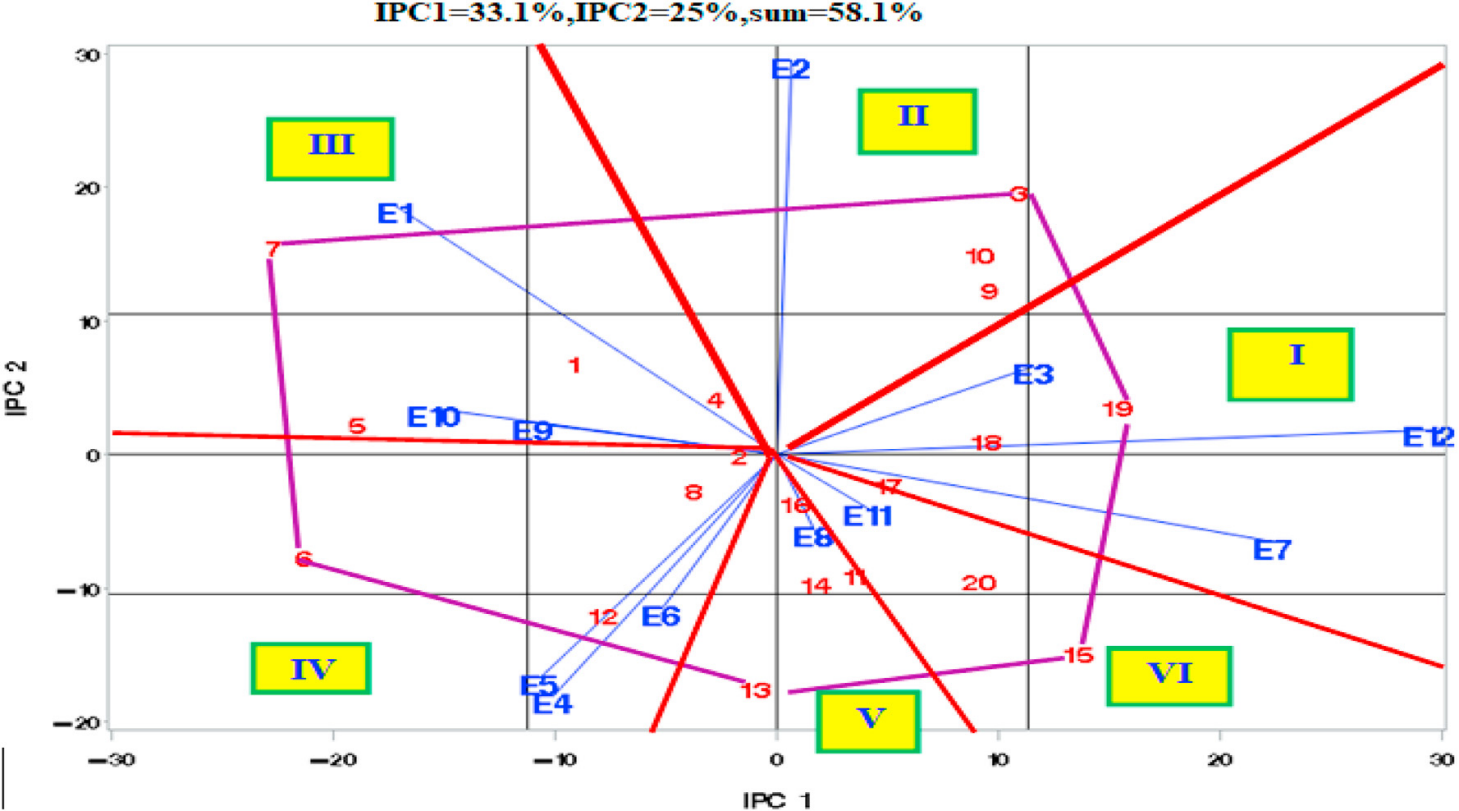
AMMI Bi-plot of IPC2 versus IPCA1.

The graph of IPCA2 versus IPCA1 revealed that the Meher, 2016 environments (E1, E2, and E3) had positive IPC2 values, while the Meher, 2017 environments (E4, E5, and E6) had negative IPC2 values (Figure 2). Belg environments (E7 to E12) had near-zero IPC2 values. IPC2 seems to be connected with the Meher of the two years, 2016 and 2017. IPCA1 can be considered a reflection of the productivity of an environment. All environments with a mean yield above the grand mean (E1, E4, E5, E6, E9, and E10), except E3 and E7 had negative IPCA1 values. All environments with below-average mean grain yield (E2, E8, E11, and E12) had positive IPCA1 values (Figure 2). IPCA3 has classified environments into two categories (Belg environments, E7 to E12) with positive IPCA3 values and Meher environments (E1 to E6) with negative IPCA3 values (Table 4). IPCA3, therefore, classified environments into Meher and Belg environments. The Meher season had a longer mean growing period (91 vs 81 days) and higher rainfall (682 mm vs 524 mm) as compared to the Belg season. All Kucha environments (E3, E6, and E9, except E12) had positive IPCA4 values while all Gofa environments (E1, E4, E7, and E10) had negative IPCA4 values. The Humbo Environments (E2, E5, E8, and E11) had near-zero IPCA4 values (Table 4). IPCA4 is, thus a contrast of Gofa with Kucha (Table 4).

**Table 4:** Mean yield (t ha^−1^), ASV, GSI of 20 cowpea genotypes tested at 12 environments, and principal component analysis of the AMMI.

Code	YD	RYD	ASV	RASV	GSI	RGSI	E	IPCA1	IPCA2	IPCA3	IPCA4	F-value
G1	0.931	19	14.1	9	28	15	E1	-17.2	18.7	-7.3	-9.3	12.6
G2	1.363	10	2.2	1	11	3	E2	0.7	29.4	-1.3	-6.7	10.1
G3	1.384	8	24.7	17	25	14	E3	11.5	6.6	-20.5	20.7	12.6
G4	1.389	7	5.9	4	11	3	E4	-10.1	-18.1	-8.3	-17.1	9.9
G5	1.311	13	25.2	18	31	17	E5	-10.7	-16.7	-0.1	1.6	17.0
G6	1.323	12	29.2	19	31	17	E6	-5.2	-11.5	-11.5	4.6	4.7
G7	1.326	11	34.0	20	31	17	E7	22.4	-6.5	5.0	-5.8	14.8
G8	1.015	18	5.5	3	21	9	E8	1.6	-5.5	0.5	4.0	7.0
G9	0.920	20	18.1	13	33	20	E9	-11.0	2.3	23.0	13.6	16.4
G10	1.376	9	19.7	14	23	11	E10	-15.5	3.4	6.9	-1.2	12.3
G11	1.045	17	9.8	7	24	12	E11	4.1	-4.0	8.2	5.7	8.9
G12	1.669	1	15.6	11	12	5	E12	29.4	1.9	6.2	-10.0	25.0
G13	1.506	5	17.1	12	17	7						
G14	1.554	3	9.6	6	9	2						
G15	1.453	6	23.1	16	22	10						
G16	1.618	2	3.5	2	4	1						
G17	1.099	15	7.0	5	20	8						
G18	1.062	16	12.6	8	24	12						
G19	1.175	14	20.8	15	29	16						
G20	1.512	4	15.6	10	14	6						
GM	**1.302**		**15.6**		**21.0**							

YD = yield, RYD = Rank by Yield; ASV = AMMI stability value; RASV = Rank by ASV; RGSI = Rank by Genotype Selection Index; GM = Grand mean, E = Environment.

##### 3.3.2.1 GEI patterns of environments and genotypes

The plot of IPCA2 vs IPCA1 has divided the environments into six sectors (Figure 2). Environments within the same sector had a small angle (<90^0^) between themselves and had a high positive correlation with each other. Genotypes at the vertex of a sector had the highest positive GxE interaction with environments in that sector and the largest in absolute value negative GEI with environments on the opposite side (at 180^0^ with them). Environments at 90^0^ from each other are uncorrelated while an angle of > 90^0^ indicates a negative correlation between the environments.

**Sector I** had E3 (Kucha, Meher, 2016), E12 (Kucha, Belg, 2018), and E7 (Gofa, Belg, 2017). G19 had the highest positive GxE interaction with these environments. **Sector II** contained E2 (Humbo, Meher, 2016). G3 had the highest positive interaction with E2. **Sector III** contained E1 (Gofa, Meher, 2016), E9 (Kucha, Belg, 2017), and E10 (Gofa, Belg, 2018). **Sector IV** had E4, E5, E6 (Meher, 2017 environments of Gofa, Humbo, and Kucha). G6 had a maximum positive interaction with these environments. **Sector V** contained E8 (Humbo, Belg, 2017) with G13 at its vertex. **Sector VI** contained E11 (Humbo, Belg, 2018) and G15 had the most favorable interaction with this environment. The angle between environments of **Sector I** (E3, E7, and E12) and **Sector** VI (E11) was narrow and these environments were correlated and had similar GEI patterns. This correlation ranged from r = 0.33 (between E3 and E11) to r = 0.89∗∗∗ (between E7 and E11). Environments of sector I except E3 were also positively correlated with E8 (sector V).

The underlying causes of the interaction observed may be due to the genetic differences between the genotypes (phenology, growth habit, predominant yield components, disease resistance, etc.) and the difference in the environments in edaphic factors, in seasonal rainfall and temperature, in the length of the growing season, in periods at which moisture stress occurred, and prevalence of the disease.

G6 and G12 had a large positive interaction with E4, E5, and E6, but large negative interaction with E2, E3, E7, and E12. These two genotypes were relatively late maturing (DF of 50 and 48 and DM of 91 and 89 days). The mean grain filling period of G12 and G6 under environments with longer growing seasons was 51 days. This was reduced to 39 and 42 days, respectively, under shorter growing seasons (E2, E3, E7, and E12), where they could not fill their grains properly (results not shown).

On the contrary, genotypes G3, G9, G10, and G19 had opposite responses to the two groups of environments as they had large positive GxE interaction with environments that had a short growing season (E2, E3, E7, and E12), but large negative interaction with environments that had a long growing season (E4, E5, and E6). These genotypes were relatively early maturing (DF of 46 days and DM of 86 days and G9 with DM of 83 days). They might have been exposed to various diseases during the grain filling period under long rainy seasons. There was a negative correlation between the environments of sector VI (G4, G5, and G6) on one side and environments of sectors I and II (ranging between -0.19 (E7 with E6) to -0.91∗∗∗ (E2 and E6).

Genotypes G5 and G7 had a positive interaction with E1, but they had a negative interaction with E7, E8, and E11. These were late-maturing genotypes (DM of 92 and 91 days). The mean growing season of E1 was 91 days while the mean growing seasons of E7, E8, and E11 were 80, 82, and 82 days, respectively. These genotypes could not fill their grains during these very short growing seasons and therefore had a negative interaction with these environments. On the contrary, G15 and G13 (DM of 84 and 85 days) interacted positively with E7, E8, and E11 but negatively with E1. The positive interaction of G5 and G7 and the negative interaction of G13 and G15 with E9 and E10 can be explained by days to flowering. Genotypes that flowered very early, much earlier than the average DF at E9 (46 days) and E10 (43 days) seems to have been exposed to pre-flowering moisture stress and interacted with them negatively. G13 and G15 flowered very early at E9 (43 days) and E10 (39 days) and were at disadvantage and hence interacted negatively with these environments while G5 and G7 flowered late at E9 (44, 48 days) and E10 (44 days) and had an advantage at these environments and interacted positively with them. The late maturity of these genotypes at E9 and E10 did not lead to negative interaction, probably because these genotypes had deep roots and have avoided moisture stress during maturity. A deep root system in cowpea could be an advantageous method to cultivate in areas where water deficits (Matsui and Singh, 2003; Yuka et al., 2019). Response of G12 and G13 at E9 and E10 were opposite. G12 interacted positively while G13 interacted negatively; G12 flowered later at these environments while G13 flowered very early and was exposed to moisture stress at flowering (results not presented). Their response to E4, E5, and E6 was the same (positive GEI). G5, G6, G7, G12, and G13 all interacted negatively with E7. However, G15 interacted positively with this environment.

##### 3.3.2.2 Grouping genotypes by stability and performance

Genotypes within one standard deviation of both IPCA1 and IPCA2 were designated as stable and those outside this range as unstable (Figure 2). Environments and genotypes close to the origin of the biplot had made a minimum contribution to GEI SS. G1, G2, G4, G8, G11, G14, G16, G17, G18, and G20, each contributed less than 5.2% to GEI SS. Cumulatively they have captured 33.5% of GEI SS (3.4% per genotype). They were the most stable genotypes and of these only G2, G4, G14, G16, and G20 (25% of the tested Genotypes) were among the high-yielding genotypes and ranked 10^th^, 7^th^, 3^rd^, 2^nd,^ and 4^th^ by mean yield over 12 environments (Table 5). G1, G8, G11, G17, and G18 gave low yields and were undesirable, although they were stable. E3, E6, E8, E9, E11, and E10 had the shortest vectors and were near the center of the biplot, capturing from 3.6 to 9.1%, and cumulatively 35.8% of GEI SS (6.0% each).

**Table 5:** Productivity and Stability (within one STD of IPCA1 and IPCA2) of Genotypes.

Stability	Yield	
	High	Low
Stable	G2, G4, G14, G16, G20	G1, G8, G11, G17, G18
Unstable	G3, G5, G6, G7, G10, G12, G13, G15	G9, G19

Environments and genotypes furthest from the origin such as E1, E4, E7, E2, E5, and E12, and genotypes G5, G6, G7, G3, G10, G9, G12, G13, G15, and G19 made the largest contribution to GEI SS (Figure 2). These ten genotypes were unstable and captured 66.6% of GEI SS (6.7% each). All, except G9 and G19, were high-yielding. The six environments (E1, E4, E7, E2, E5, and E12) captured from 6.8 to 14.2% and cumulatively 64.1% of GEI SS (10.7% each). These are the most discriminating environments. Other environments were also discriminating in the sense that the F-test

for Genotype Mean Square was very highly significant at all 12 environments. The F-value varied from 4.69 (E6) to 25.0 (E12) with the F-required at a probability level of 0.001 to be 3.24. The 20 genotypes can be classified by yield and stability into four groups (Table 5).G3, G5, G6, G7, G10, G12, G13, and G15 ranked 8^th^, 13^th^, 12^th^, 11^th^, 9^th^, 1^st^, 5^th,^ and 6^th^ by average yield over 12 environments and need further evaluation to identify environments to which they are specifically adapted especially G3, G10, G12, G13, and G15.

### 3.4 AMMI stability value (ASV) and genotype selection index (GSI)

ASV ranged from 2.2 for variety Bole (G2) to 34 for genotype Brazil-4 (G7) (Table 5). AMMI stability value (ASV) discriminated genotypes G2 (2.2), G16 (3.5), G8 (5.5), G4 (5.9), G17 (7.0), G14 (9.6), and G11 (9.8), as the stable genotypes, respectively (Table 4). Genotype Selection Index (GSI) discriminated G16 (4.0), G14(9), G4(11), and G2 (11) with general adaptability and high grain yield for belg and meher seasons conditions which were in agreement with the results of biplot analysis. Stability per se should however not be the only parameter for selection, because the most stable genotypes would not necessarily give the highest yield (Mohammadi and Amri, 2008); so there is a need for approaches that incorporate both mean grain yield and stability in single criteria. In this regard, Farshadfar (2008) proposed Genotype Selection Index (GSI) as a selection criterion to identify the most desirable genotypes, stable genotypes with high yield. The least GSI is considered as the most stable with a high grain yield (Table 4). The ASV and GSI indicators have been applied by numerous researchers (Olayiwola and Ariyo, 2013; Horn et al., 2018; Farshadfar, 2008; Mohammadi and Amri, 2008).

### 3.5 GGE biplot analysis

**Figure 3:**
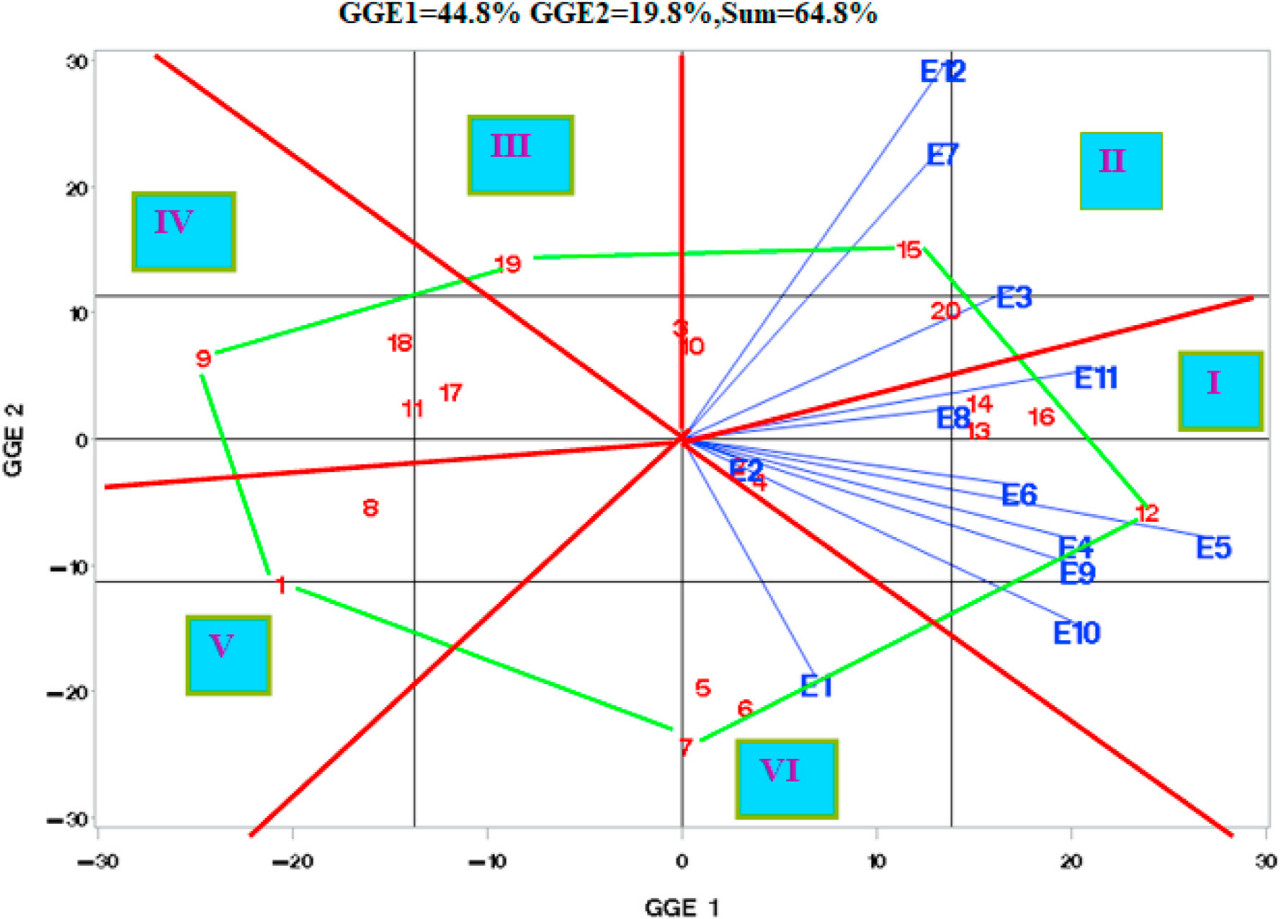
The plot of GIPCA2 vs GIPCA1 for 20 cowpea genotypes tested at 12 Environments.

The GGE biplot analysis of grain yield response and stability of 20 cowpeas showed that the first three components accounted for 44.8, 19.8, and 12.7 % of the GGE sum of squares, respectively, explaining a total of 77.3 % of the SS and 40.2% of the DF of GGE (Figure 3). Therefore, GGE-III seems to be appropriate to explain the complex GGE of 20 cowpea genotypes tested at 12 environments in our study. The remaining nine components explained from 0.8 to 6% of GEI SS (eight of them captured <5% of GEI SS each). This suggested that the biplot of PC1, PC2, and PC3 adequately approximated the environment-centered data. The results disagree with the previous research results which showed the first two principal components to be adequate to explain the GEI (Matova and Gasurae, 2018; Horn et al., 2018). The plot of the first principal component (GIPCA1) versus the second principal component (GIPCA2) revealed that GIPCA1 ranked genotypes according to their mean yield, i.e., GIPCA1 represents G. This is witnessed by the distribution of the 20 genotypes along the GIPCA1 axis (rank correlation by GIPCA1 and actual yield was 0.95∗∗∗). GIPCA2 (19.8%) should then be the expression of GEI.

GIPCA2 has divided the environments into those with no or minimal terminal moisture stress (E1, E2, E4, E5, E6, E9, and E10) with negative GIPCA2, and environments with severe terminal moisture stress (E3, E7, E8, E11, and E12) with positive GIPCA2 (Figure 3). GIPCA3 (12.7%) seems to show the comparison of seasons within a year; comparison of Meher seasons in 2016 and 2017 and comparison of Belg seasons of 2017 and 2018 (only E9 is misplaced) (results not shown).

For this investigation, the polygon view (Figure 3) showed the “which-won-where” pattern of the ME trials data. The fitted GGE-biplot model indicated that the first two PCs explained 64.6 % of the variation for G + GE (Figure 3). The polygon view of the GGE biplot was drawn by connecting the vertices genotypes with straight lines so that all other genotypes were contained within the polygon. In GGE-2 vs GGE-1 biplot, red lines arising from the origin and perpendicular to the lines connecting the vertex genotypes G12, G15, G19, G9, G1, and G7, have divided the environments and genotypes into six different sectors (Figure 3). The more favorable Meher environments, E4, E5, and E6, with E2, E9, and E10 on one side and E8 and E11 on the other side joining them, formed Sector I. E3, E7, and E12 formed a separate sector (Sector II) as in AMMI. In the GGE-biplot, E1 is separated from E9 and E10 and is placed alone in Sector VI.

Environments in the same sector had similar GEI patterns. The correlation between E1 and environments of the sector I ranged between 0.21 (E1 with E11) to 0.81∗∗∗ (E1 with E10). E1 can be considered as uncorrelated with E8 and E11 and as belonging to a different mega-environment. E1 in sector VI was negatively correlated with all environments of Sector II and the two sectors can be considered to belong to different mega-environments. We, therefore, had three sectors, which can be considered to be three mega-environments; Sector I, with E2, E4, E5, E6, E8, E9, E10, and E11, sector II with E3, E7, and E12, and sector VI with E1.

G12 was the winning genotype in Sector I, while G15 and G7 were the winning genotypes in Sectors II and VI, respectively. However, G16 in sector I, G20 in sector II, and G5, and G6 in sector VI were also near the vertices and gave high yield in their respective sectors.

There were no environments in sectors III, IV, and V. All genotypes of these sectors (G9 (BEB), G11 (local check), and advanced lines G1, G8, G17, G18, and G19, gave below-average yields at all environments, except G19 at E2, E3, E7, and E12, G1 at E1 and G18 at E7 and E12 (Figure 3). When genotypes give rise to vertices of polygons but do not contain any environment clustered in their respective sector, they are considered un-adapted to all test environments (Miranda et al., 2009) and G9 was such a genotype.

Ranks of the highest yielding genotypes by mean grain yield estimated from GIPCA1, GIPCA2, and GIPCA3 were G12, G16, G14, G20, G13, G15, G4, G3, G10, G2, G7, G6, and G5. The late-maturing genotypes, G5 (Brazil-2), G6 (Brazil-3), and G7 (Brazil-4) gave high yield at E1, E2, E4, E5, E6, E9, and E10 (except G6 at E2 and G7 at E4 and E6) but low yield at E3, E7, E11, and E12 where they matured very late and could not fill their grain properly. All three late-maturing genotypes performed best at E1 where they ranked 2^nd^, 3^rd,^ and 1^st^, respectively. G5 and G7 ranked 6^th^ and 2^nd^ at E2. These advanced lines were not the highest yielding under the favorable Meher environments, E4, E5, and E6, where they were very late. However, G6 ranked 6^th^, 3^rd^, and 4^th^ at E4, E5, and E6 and can be recommended for these favorable environments with a long growing period (100–110 days). G5, G6, and G7 also ranked 2^nd^ to 7^th^ at E9 and E10. All three can, therefore, be recommended for Meher seasons with the intermediate length of the growing period (91 days) and Belg seasons with a short growing period (77–82 days) but with no terminal moisture stress. G5 and G7 can also be recommended for Meher seasons with a short growing period (85 days) similar to E2.

The early-maturing varieties, G3 (White wonderer trailing) and G10 (TGVU-1977-DD1) had an opposite response as compared to G5, G6, and G7. They performed well at E3, E7, E11, and E12 but performed poorly at E4, E5, and E6, where they matured much earlier and might have been exposed to diseases during the grain-filling period. G3 ranked 1^st^ at E2 and E3 while G10 ranked 3^rd^ at both environments. They were 3^rd^ and 4^th^ at E12, 6^th^ and 8^th^ at E1, and 7^th^ and 8^th^ at E11, respectively. They can be recommended for Meher and Belg environments with short growing period and with terminal moisture stress such as E1 (91 days), E2 (85days), E3 (87 days), E7 (80 days), E11 (82 days), and E12 (79 days). The relatively early maturing advanced lines, G15 (IT99K-1060) and G20 (IT97K-569-9) also performed best at environments with severe moisture stress during the grain-filling period such as E7 (80 days) and E12 (79 days), where G15 was 1^st^ and G20 was 2^nd^. They also ranked 3^rd^ to 7^th^ at all remaining environments except at E1, E2, E9, and E10, where they were exposed to pre-flowering moisture stress. These advanced lines can, therefore, be recommended for all Meher and Belg environments, avoiding those with intermediate growing periods such as E1 and E2 and those with pre-flowering moisture stress but with no terminal moisture stress, such as E9 and E10.

G12 and G16 gave high yields at all 12 environments while G13, G14, G15, and G20 also gave high yields at all environments except at E1 and E2. G2 also gave an above-average yield at all environments except at E12. G4 gave a below-average yield at only E4 and E7. Ranks of G16 (2^nd^ to 7^th^) were more constant than those of G12 (1^st^ to 9^th^). Therefore advanced line G16 (IT-89KD) can be recommended for all cowpea growing areas in southern Ethiopia. G12 was the highest yielding genotype (1^st^) in seven environments; under Meher seasons with a long and favorable growing period (100–110 days) such as E4, E5, and E6 and under Belg environments with no terminal moisture stress such as E9 and E10 and it also ranked 1^st^ at E11. For G12 Meher and Belg environments with short growing periods and with terminal moisture stress such as E2 (85 days) and E12 (80 days), where it ranked 9^th^ should be avoided. G13 and G14 can also be recommended for all cowpea growing Meher and Belg environments avoiding Meher seasons with a short and intermediate growing period such as E1, E2, and E3 and Belg seasons with a very short growing period such as E12. The released variety G4 performed well under E1 and E2, which represent Meher seasons with a short and intermediate growing period where it ranked 5^th^ and 4^th^.

#### 3.5.1 Mean yield and stability

Yield and stability of genotypes were estimated using the Average Environment Coordinate (AEC), which is defined by a line passing through the origin of the bi-plot and the average of GIPC1 and GIPC2 scores (Yan and Kang, 2003). The best genotype (ideal genotype) can be defined as the one with the highest yield and highest stability across environments. Genotypes with high GIPC1 scores have high mean yield and those with low (in absolute value) GIPC2 scores have stable yield across environments (Yan and Tinker, 2006). A longer projection to the AEC ordinate, regardless of the direction, represents a greater tendency of the GxE interaction of a genotype, which means that its performance is more variable and the genotype is less stable across environments or vice versa.

**Figure 4:**
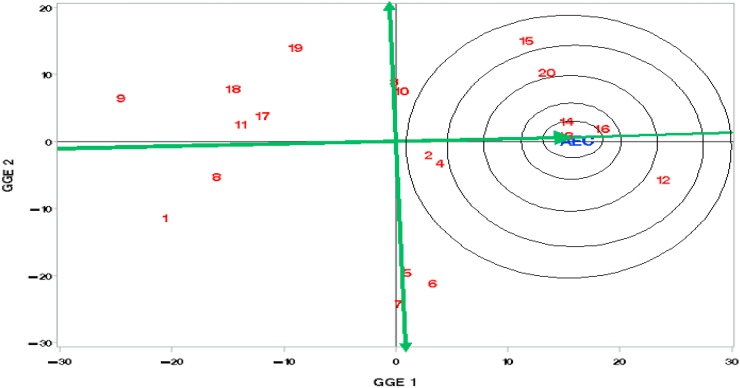
Comparison of genotypes with an ideal genotype.

The line perpendicular to the average environment coordinate is represented as a double-headed arrow and points towards lower stability in both directions (Figure 4). Genotypes on the left side of the ordinate line (with double arrows) had yields less than the mean yield while those on its right had above-average yield. G13 (93K-619-1) at the center of the first concentric circle, was the most desirable genotype for grain yield and stability followed by G14, G16, and G20, which are located in the subsequent concentric circles (Figure 4). The ideal genotype can be used as a reference for evaluating genotypes and identifying stable and high-yielding genotypes for wide adaptation. A genotype is desirable if it is closer to the ideal genotype (Yan and Hunt, 2002).

Genotypes G2, G4, G13, G14, and G16 were high-yielding and stable, suggesting their adaptation to a wide range of environments, the most desirable situation for plant breeders (Figure 4, Table 6). G3, G10, G5, G6, G15, G20, and G12 were high-yielding but unstable, which can also be considered desirable. Genotypes G9, G8, G11, and G17 were stable but at the low-yielding-3^rd^ level in desirability. G1, G18, and G19 were low-yielding and unstable (fourth in desirability, i.e., most undesirable) (Table 6). This classification is similar to the one achieved by using the distance of the genotype from the center of the IPCA1-IPCA2 biplot of AMMI (Table 5).

**Table 6:** Classification of Genotypes by Desirability based on rank by GGE1 and GGE2 (GEI).

Desirability	Classification by Yield and Stability	Genotypes
1. Most Desirable	High yielding and Stable	G13, G16, G14, G2, G4
2. Desirable	High yielding and Unstable	G12, G20, G10, G15, G3, G6, G5, G7
3. Undesirable	Low yielding and Stable	G11, G17, G8, G9
4. Most undesirable	Low yielding and Unstable	G18, G19, G1

## 4 Conclusions and recommendations

This study indicated that the effect of genotype, environment, and their interaction were highly significant for grain yield of cowpea genotypes tested during Meher and Belg growing seasons in Southern Ethiopia. The polygon views of the GGE biplot pointed out that there existed three possible mega-environments. The study also confirmed that higher yields were realized at Gofa than Humbo during the study period.

Various stability models were used in the measurement of genotype stability such as AMMI Stability Value (ASV), GSI, and GGE. Grouping genotypes by Yield, ASV, and GSI gave G2, G4, G12, G14, G16, G20 as stable and high-yielding genotypes. Biplot of GEIPCA2 vs GEIPCA1 produced G13, G16, G14, G2, G4 as stable and high-yielding genotypes. G5, G6, and G7 were also average-yielding, but inconsistent and thus can be recommended for Meher environments with the intermediate length of the growing period (91 days) and Belg seasons with no terminal moisture stress such as E9 and E10.

AMMI and GGE biplot, ASV, and GSI indices identified G16 and G14 genotypes present the highest yielding with better stability across environments and had higher grain yields than the checks, suggesting that it can be recommended for all cowpea growing areas of southern Ethiopia with soil and weather conditions similar to the areas used in this study. The advanced lines G12, G13, G20, and G15 had higher grain yields than the checks and were suggested for further inclusion in breeding to boost cowpea production.

## Declarations

### Author contribution statement

Yasin Goa: Conceived and designed the experiments; Performed the experiments; Analyzed and interpreted the data; Wrote the paper.

Hussein Mohammed: Conceived and designed the experiments; Analyzed and interpreted the data; Wrote the paper.

Walelign Worku: Conceived and designed the experiments; Performed the experiments; Wrote the paper.

Elias Urage: Performed the experiments; Wrote the paper.

### Funding statement

This research did not receive any specific grant from funding agencies in the public, commercial, or not-for-profit sectors.

### Data availability statement

Data included in article/supplementary material/referenced in article.

### Declaration of interests statement

The authors declare no conflict of interest.

### Additional information

No additional information is available for this paper.
